# Etymologia: Verona Integron

**DOI:** 10.3201/eid1907.ET1907

**Published:** 2013-07

**Keywords:** etymologia, Verona integron, integron, Pseudomonas aeruginosa, antibiotic resistance, antimicrobial resistance, bacteria

**Verona Integron**

From the Latin *integrare* (to make whole), integrons are systems for capturing and spreading antibiotic resistance genes among gram-negative bacteria. Integrons were first described by Stokes and Hall in 1989, although they clearly contributed to the first outbreaks of multidrug resistance in the 1950s. The Verona integron was first described in carbapenem-resistant *Pseudomonas aeruginosa* isolated from a patient hospitalized at Verona University Hospital, Verona, Italy. Integrons are ancient structures that have been present in bacteria for millions of years, indicating that bacteria had the means of acquiring and disseminating antibiotic resistance long before humans developed antibiotics.
